# Evaluating the Supporting Evidence of Medical Cannabis Claims Made on Clinic Websites: Cross-Sectional Study

**DOI:** 10.2196/45550

**Published:** 2023-06-29

**Authors:** Braden O'Neill, Jacob Ferguson, Lauren Dalueg, Abban Yusuf, Abirami Kirubarajan, Taryn Lloyd, Eisi Mollanji, Navindra Persaud

**Affiliations:** 1 Department of Family and Community Medicine St. Michael’s Hospital Unity Health Toronto Toronto, ON Canada; 2 Temerty Faculty of Medicine University of Toronto Toronto, ON Canada; 3 University College Dublin Dublin Ireland; 4 MAP Centre for Urban Health Solutions St. Michael's Hospital Unity Health Toronto Toronto, ON Canada; 5 Department of Obstetrics and Gynaecology Faculty of Health Sciences McMaster University Hamilton, ON Canada; 6 Department of Emergency Medicine St. Michael's Hospital Unity Health Toronto Toronto, ON Canada; 7 Faculty of Medicine University of Ottawa Ottawa, ON Canada

**Keywords:** cannabis, evidence-based medicine, adverse effects, consumer health information

## Abstract

**Background:**

Since the legalization of medical cannabis in Canada in 2013, prescription of cannabis for medical purposes has become commonplace and a multibillion dollar industry has formed. Much of the media coverage surrounding medical cannabis has been positive in nature, leading to Canadians potentially underestimating the adverse effects of medical cannabis use. In recent years, there has been a large increase in clinic websites advertising the use of medical cannabis for health indications. However, little is known about the quality of the evidence used by these clinic websites to describe the effectiveness of cannabis used for medical purposes.

**Objective:**

We aimed to identify the indications for medical cannabis reported by cannabis clinics in Ontario, Canada, and the evidence these clinics cited to support cannabis prescription.

**Methods:**

We conducted a cross-sectional web search to identify all cannabis clinic websites within Ontario, Canada, that had physician involvement and identified their primary purpose as cannabis prescription. Two reviewers independently searched these websites to identify all medical indications for which cannabis was promoted and reviewed and critically appraised all studies cited using the Oxford Centre for Evidence-Based Medicine Levels of Evidence rubric.

**Results:**

A total of 29 clinics were identified, promoting cannabis for 20 different medical indications including migraines, insomnia, and fibromyalgia. There were 235 unique studies cited on these websites to support the effectiveness of cannabis for these indications. A high proportion (36/235, 15.3%) of the studies were identified to be at the lowest level of evidence (level 5). Only 4 clinic websites included any mention of harms associated with cannabis.

**Conclusions:**

Cannabis clinic websites generally promote cannabis use as medically effective but cite low-quality evidence to support these claims and rarely discuss harms. The recommendation of cannabis as a general therapeutic for many indications unsupported by high-quality evidence is potentially misleading for medical practitioners and patients. This disparity should be carefully evaluated in context of the specific medical indication and an individualized patient risk assessment. Our work illustrates the need to increase the quality of research performed on the medical effects of cannabis.

## Introduction

A multibillion dollar cannabis industry has emerged in Canada after its medical use was legalized in 2013 and recreational use was legalized in 2018 [1]. Before 2018, authorized medical cannabis was procured from federally licensed producers under the Access to Cannabis for Medical Purposes Regulations [2-4]. After recreational legalization, federally licensed producers in Ontario revoked their medical designations, as any consumer, medical or recreational, could obtain their cannabis from any licensed dispensary, in addition to growing or producing it themselves [2-4]. After 2018, “medical cannabis” could be any cannabis product, including the same products used recreationally but obtained with a medical indication. Overall, these changes have also fostered societal shifts in cannabis use among some segments of the population; for instance, among adolescent males, substance-related hospitalizations after cannabis legalization have increased 30% in Quebec [5]. Modest increases in cannabis use among middle- and older-aged adults have also been seen after legalization, even though regulated cannabis generally costs more than illegally obtained cannabis [6,7]. Despite cannabis being medically marketed, it is still not considered an “approved health product” by the Government of Canada [8]. Although Health Canada maintains a system for reporting adverse events associated with cannabis products on its “Canada Vigilance” website, it is not mandatory for health care providers to make a report, although it is for licensed cannabis producers [9]. The cannabis industry’s rapid expansion has raised concerns that the health implications of cannabis legalization have been insufficiently evaluated; some experts have argued that further regulation of medical cannabis and cannabis-prescribing clinics is critical to mitigating possible harms and injuries to patients across Canada [10]. In general, promotion of cannabis in the media has been shown to overestimate its beneficial effects, such as limiting illegal sales of cannabis, lowering costs related to cannabis policing and court work, and increasing tax revenue [7,11]. Moreover, recent media and testimonials in Canada surrounding the benefits of medical cannabis tend to skew overwhelmingly positive [10], leading to many Canadians underestimating potential harms and how to mitigate them and overestimating the safety of cannabis-containing health products [12,13]. For instance, Health Canada [12] found that only 10% of overall Canadians and 13% of cannabis users in Canada were aware of the Lower-Risk Cannabis Use Guidelines, which describes key harms of cannabis (such as sedation and confusion) and how best to avoid them [14].

Cannabis has long been proposed to negatively impact cognitive development among youths who frequently use it [5,7], leading to a recent call for improving the regulation of medical cannabis in Canada, specifically for the health of pediatric patients [15]. Similarly, excessive cannabis use may be a concern for older populations, where worries regarding the development of cannabis use disorder and other cannabis-related adverse effects led to the development of the Canadian Guidelines on Cannabis Use Disorder Among Older Adults [16]. It is possible that because many medical conditions have higher prevalence in older adults, they are more likely to seek out cannabis for medical purposes.

There is a paucity of high-quality evidence demonstrating the effectiveness of cannabis for medical purposes [10,17-19]. A recent systematic review on the use of medical cannabis found some low- to moderate-quality evidence to support its use for nausea and vomiting after chemotherapy, for spasticity from multiple sclerosis, and for neuropathic pain, but no high-quality evidence supporting its use for other indications, such as osteoarthritis pain [17]. Adverse events from cannabis use are common, such as dizziness, confusion, and sedation [17]. Further, rates of cannabis hyperemesis syndrome, lung injury, arrhythmia, and cannabis-related trauma are all causes of increasing emergency department use [14-17,20].

Despite this uncertainty in the evidence base, there has been an explosive proliferation of cannabis clinics and physicians prescribing cannabis throughout Ontario [21]. Most of the medical expansion occurred before outright recreational legalization. However, within Canadian and Ontarian law, there are differences in the regulation and taxation of medical and recreational cannabis. Recreational cannabis can be purchased at retail stores known as *dispensaries*, whereas medical cannabis must be prescribed to a patient at a clinic. Persons with medical indications are permitted to publicly carry more than the allotted recreational 30 g limit (up to 150 g). Medical cannabis growers can also grow more plants. Further, medical cannabis is subject to lower tax rates than recreational cannabis, resulting in lower direct fees to the consumer, and spending on medical cannabis also qualifies as an income tax deductible expense [3].

Many of these publicly accessible clinic websites make claims of the effectiveness of cannabis for various health issues [10]. The web is a widely used health information resource; a nationally representative survey found that two-thirds of Canadians looked for health information on the web in the past year [22]. People may also evaluate medical cannabis websites to better understand health benefits and risks, whether using recreationally or as an intended medical therapeutic. Social assistance programs, such as the Ontario Drug Benefit program, offer compassionate programming or compensation on medical cannabis products such as nabilone [23] to adults older than 65 years and those receiving support from the Ontario Disability Support Program [24]. Several large insurance companies in Canada also offer coverage for medical cannabis if prescribed by a physician, albeit for a limited number of conditions, such as rare types of epilepsy [25-27]. There are also protected human rights within employment law affording unique protections around employment if using medical cannabis when recreational cannabis would not be tolerated.

The purpose of this study was to systematically assess the evidence supporting claims of cannabis effectiveness for various indications, as found on cannabis prescribers’ websites. We analyzed the breadth and quality of the research cited by these clinics for the conditions for which cannabis is being promoted, and we describe the extent to which potential harms and side effects of cannabis are mentioned alongside its promotion.

## Methods

### Study Design

We adapted a cross-sectional methodology that members of our team used previously to systematically identify and assess claims of effectiveness of sports performance products [28] in order to identify claims related to cannabis and assess the quality of supporting evidence. Our independent variables were the websites of cannabis clinics in Ontario, whereas our dependent variable was the supporting evidence provided for health indications that were reported on each site. We then performed a systematic assessment of the supporting evidence to ascertain the quality of the evidence supporting claims of the effectiveness of medical cannabis for various health indications.

We conducted web searches to identify cannabis clinics in Ontario, Canada. City locations of all clinics were recorded to ensure that they were located within Ontario. The province of Ontario was chosen as a sample because it is the most populous Canadian province, with approximately 15 million residents [29]. In some cases, there was a parent company that owned multiple clinics across Canada. When this occurred, we looked through available pages on the parent company website to identify that there was a physical clinic location in Ontario. Then, we analyzed data from the parent company website. The medical claims and cited supporting evidence included were not limited to Ontario, and thus could be broadly applicable across the country, where cannabis has been legalized nationally. One team member conducted Google searches in August 2021 using the keywords *cannabis*, *clinic*, *medical marijuana*, *dispensary*, and *pain clinic*, combined with the OR Boolean operator. We limited searches to the top 100 hits, which has been established in other studies as an appropriate approach for web searches of this type [30]. The team member then viewed every link in the top 100 search results and independently identified whether the website demonstrated physician involvement in the prescription of cannabis for medical purposes. Clinics that did not claim to have a physician licensed to practice medicine in Ontario [31] identifiable on their website (commonly referred to as *dispensaries*) [32] were excluded. A second reviewer independently visited all identified websites and assessed for inclusion. Microsoft Excel (Microsoft Corp) was used to document search results and for data handling overall within the study, including analysis of descriptive statistics.

The 2 team members independently and in parallel reviewed each page of every clinic website and documented each health claim or condition for which cannabis was recommended on the website (eg, “cannabis is helpful for pain” and “insomnia”), as well as any evidence cited supporting the claim. Evidence cited included information directly from the website or references to external sources. We compiled all health claims, qualitatively grouped them by content and theme when possible, and determined the following: number of total clinics, number of claims per clinic, number of specific indications identified, and number of claims per specific indication. Data collected included data on quality of evidence, study characteristics, cannabinoids used, routes of cannabis administration, and strain of the cannabis plant. We also tracked which clinic websites mentioned potential harms related to medical cannabis.

Two team members independently in parallel appraised the quality of “supporting evidence” for all health claims across all cannabis clinic websites using the Oxford Levels of Evidence [33]. This is an established framework for evaluating evidence quality where a systematic review represents the highest available quality of evidence and is classified as “level 1,” a randomized controlled trial (RCT) is classified as a “level 2” study, and further study types are progressively hierarchically classified, ending with a study conducted using animal models of disease being classified as “level 5”—the lowest-quality evidence.

Discrepancies were resolved through discussion with a third reviewer. We report the overall quality of evidence as well as the quality of evidence by specific indication.

### Ethical Considerations

This study used only publicly available data and did not involve human participants and as a result, research ethics board approval was not sought or required.

## Results

### Overview of Identified Clinic Websites

**Figure 1:**
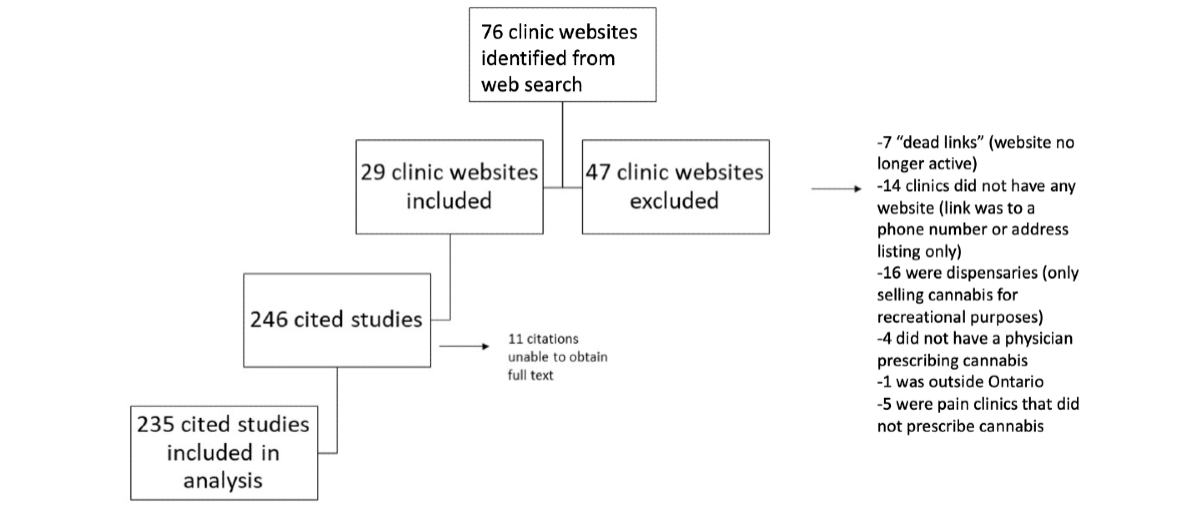
Study flow diagram.

We found 76 total potentially eligible cannabis clinic websites across Ontario from our search, of which 47 websites were excluded, yielding 29 clinics and their websites for inclusion in the study (Figure 1). Of the 47 excluded websites, 7 (15%) were links to websites that were no longer functioning and which we were unable to find after a Google search using the name of the clinic as a search term; 14 (30%) only linked to phone numbers or addresses and did not have any content beyond that; and 16 (34%) were for dispensaries for commercial and recreational distribution rather than clinics where physicians prescribed cannabis. A further 4 of 47 (9%) results did not appear to be dispensaries but still had no identifiable prescribing health care provider, 1 of 47 (2%) result was excluded for being outside of Ontario, and 5 of 47 (11%) results were excluded for not indicating explicitly whether they prescribed cannabis (these were all “pain clinics” and did not have claims of effectiveness or supporting evidence related to cannabis for evaluation).

Of the 29 clinics that met our inclusion criteria, most advertised cannabis for multiple indications (Table 1). The most frequently reported indication was pain management (n=24). Other described indications included but were not limited to anxiety (n=22), rheumatoid or osteoarthritis (n=20), epilepsy (n=19), posttraumatic stress disorder (n=20), fibromyalgia (n=17), multiple sclerosis (n=16), depression (n=15), HIV/AIDS (n=10), and cancer (n=14). Only 4 clinic websites described potential harms related to cannabis use. These were all warnings that cannabis can be an addictive substance.

The clinics’ websites referenced 246 different studies to reinforce their claims; we were able to obtain full-text records for 235 citations. These studies covered the effectiveness of cannabis for indications such as addictions, depression, diabetes, dystonia, autism, arthritis, neuropathy, fibromyalgia, spinal cord injuries, various types of cancer, and posttraumatic stress disorder (Table 2).

**Table 1:** Indications promoted on cannabis clinic websites (n=29).

Indications	Clinics, n (%)
Management of pain (chronic, back, joint, neuropathic, and pelvic)	24 (83)
Anxiety	22 (76)
Arthritis (rheumatoid and osteoarthritis)	20 (69)
Posttraumatic stress disorder	20 (69)
Sleep disorders (including insomnia)	19 (66)
Epilepsy, seizures, and Dravet syndrome	19 (66)
Fibromyalgia	17 (59)
Multiple sclerosis	16 (55)
Headaches and migraines	15 (52)
Depression	15 (52)
Symptoms of cancer	14 (48)
Nausea and vomiting (chronic and chemotherapy-induced)	13 (45)
Parkinson disease	10 (35)
Bowel disease (colitis, Crohn, and irritable bowel syndrome)	10 (35)
HIV/AIDS	10 (35)
Attention deficit disorder/attention deficit hyperactivity disorder	9 (31)
Palliative care	7 (24)
Glaucoma	6 (21)

**Table 2:** Studies cited on cannabis clinic websites for specific indications (n=235).

Indications for medical cannabis	Studies, n (%)
Management of pain (chronic, back, joint, neuropathic, and pelvic)	25 (10.6)
Cancer (and treatment side effects)	16 (6.8)
Arthritis (rheumatoid, osteoarthritis)	14 (6)
Posttraumatic stress disorder	13 (5.5)
Sleep disorders (insomnia)	12 (5.1)
Multiple sclerosis	11 (4.7)
Anxiety	10 (4.3)
Addictions and withdrawals	9 (3.8)
Crohn disease	9 (3.8)
Fibromyalgia	9 (3.8)
Breast cancer	8 (3.4)
Asthma and chronic obstructive pulmonary disease	7 (3)
Diabetes (and symptoms)	7 (3)
Dystonia	7 (3)
Epilepsy and seizures	7 (3)
Migraines and headaches	7 (3)
Spinal cord injuries	7 (3)
Autism	6 (2.6)
HIV/AIDS	6 (2.6)
Parkinson disease	5 (2.2)
Prostate cancer	5 (2.2)
Depression	4 (1.7)
Irritable bowel syndrome	4 (1.7)
Neuropathy	3 (1.3)
No specific claim	3 (1.3)
Amyotrophic lateral sclerosis	2 (0.9)
Anorexia (loss of appetite)	2 (0.9)
Brain cancer	2 (0.9)
Glaucoma	2 (0.9)
Hepatitis C	2 (0.9)
Lung cancer	2 (0.9)
Lupus	2 (0.9)
Memory and reversing aging loss	2 (0.9)
Attention deficit hyperactivity disorder	1 (0.4)
Alzheimer	1 (0.4)
Inflammation	1 (0.4)
Loss of appetite	1 (0.4)
Women’s health	1 (0.4)

### Cannabinoids

Cannabidiol (CBD) and tetrahydrocannabinol (THC) were the most common cannabinoids in cited studies. In total, 26 of 235 (19.3%) studies solely used CBD, whereas 32 of 235 (13.6%) studies solely used THC; 51 of 235 (21.7%) studies assessed both THC and CBD either in conjunction or in comparison with one another; 36 of 235 (15.3%) studies assessed either CBD and THC in combination with various other cannabinoid or cannabinoid analogs, such as nabilone, drinabant, and levonantradol; and 71 of 235 (30.2%) studies did not specify which cannabinoid was studied (Table 3).

**Table 3:** Cannabinoid, strain, and route of administration in cited studies (n=235).

Cannabinoid, strain, and route of administration		n (%)	95% CI
**Type of cannabinoid**			
	Cannabidiol	26 (11.1)	7.4-15.8
	Tetrahydrocannabinol	32 (13.62)	9.5-18.7
	Both	51 (21.7)	16.6-27.5
	Unspecified	71 (30.2)	24.4-36.5
**Strain of cannabis**			
	Sativa only	34 (14.5)	10.2-19.6
	Indica only	0 (0	
	Both		
	Unspecified	8 (3.4)	1.5-6.6
**Route of administration**			
	Smoking	76 (32.3)	26.4-38.7
	Vaporization	5 (2.13)	0.6-4.9
	Oral consumption	89 (37.8)	31.7-44.4
	Sublingual administration	8 (3.4)	1.48-6.6

### Cannabis Strains

In total, 34 of 235 (14.4%) studies used cannabis from the sativa strain; 2 of 235 (0.9%) used Sativex, a synthetic type of cannabis; 8 of 235 (3.4%) studies used cannabis from both sativa and indica strains; 1/235 (0.4%) study used cannabis from any strain; and 183 of 235 (77.9%) studies did not specify which strain they used (Table 3).

### Route of Administration

Overall, 15 of 235 (6.4%) studies reported using inhalation as a route of administration, 76 of 235 (32.3%) studies measured cannabis smoking, 101 of 235 (43.5%) studies measured ingestion of cannabis (without further specification), 8 of 235 (3.4%) studies provided cannabis sublingually, 4 of 235 (1.7%) studies used edibles, 89 of 235 (37.9%) studies had cannabis taken orally, and 65 of 235 (26.4%) studies had an unspecified method of administration (Table 3).

### Overview of Cited Evidence From Clinic Websites

Of the 235 total studies cited, most were reviews (53/235, 22.6%) or surveys (40/235, 17%) (Table 4). In total, 24 of 235 (10.2%) were RCTs (Table 5). The RCTs included 2083 participants, with study sample sizes between 8 and 630 (median 41, IQR 23-68; mean 116, SD 196). Only 4 of 24 (17%) RCTs had a clear hypothesis. Most of the studies had both a control group and randomization, but only 7 of 24 (29%) used allocation concealment. Of the 24 RCTs, 9 (38%) used intention-to-treat analysis. In total 13 of 24 (54%) RCTs used blinding, but only 9 of 24 (38%) were double-blinded.

**Table 4:** Distribution of types of studies cited by cannabis-prescribing clinics (n=235).

Type of study	Studies, n (%)
Review	53 (22.6)
Survey	40 (17.0)
Basic science study	29 (12.3)
Observational cohort study (prospective and retrospective)	27 (11.5)
Randomized controlled trial	24 (10.2)
Systematic review/meta-analysis	19 (18.1)
Clinical trial (“noncontrolled” or nonrandomized)	17 (7.2)
Cross-sectional study	10 (4.3)
Case series/report	8 (3.4)
Expert opinion	6 (2.6)
Qualitative study	2 (0.9)

**Table 5:** Quality of included studies (n=235).

Level of evidence^a^	Studies, n (%)
Level 1	7 (3)
Level 2	30 (12.8)
Level 3	45 (19.1)
Level 4	63 (26.8)
Level 5	36 (15.3)
Unable to classify (narrative reviews)	54 (22.9)

^a^Level 1 studies represent the highest level of evidence and are reserved for systematic reviews; a randomized controlled trial is classified as a level 2 study; nonrandomized, cross-sectional and cohort studies are classified as level 3; level 4 studies are case-series or case-control studies; and studies conducted using animal models of disease are classified as level 5—the lowest-quality evidence.

Among the 235 total studies, there were 17 (7.2%) “noncontrolled” or nonrandomized trials. These included 1539 participants, with study sample sizes between 8 and 630 (median 20, IQR 10-47; mean 86, SD 132). Of the 17 studies, 4 (24%) had a clear hypothesis, 9 (53%) had a control group, 3 (18%) used allocation concealment, and 6 studies (35%) used intention-to-treat analysis. All 5 of the 17 studies (29%) that used blinding were reported to be double-blinded.

Of the 235 total studies, 27 (11.5%) cohort studies were cited. These included a total of 1,199,353 participants (range 20-800,000; median 188, IQR 61-655, mean 47,974, SD 167,426). In total, 3 of 27 (11.1%) studies had a clear hypothesis, 8 of 27 (29.6%) studies had a control group, 1 of 27 (3.7%) studies used allocation concealment, 4 of 27 (14.8%) studies used intention-to-treat analysis, 3 of 27 (11.1%) studies used blinding, and only 1 of 27 (3.7%) studies was double-blinded.

Most studies (218/235, 92.8%) studies enrolled adult participants 18 years and older. These studies included 1,311,211 participants in total (median 128, IQR 31-469; mean 11,019, SD 77,725). A total of 16 of 235 (6.9%) studies enrolled pediatric participants. These studies had a total study population of 412 participants (median 54, IQR 22-66; mean 69, SD 57). Of the 235 studies, 1 study (0.4%) included both adult and pediatric participants.

### Evaluation of Quality of Evidence

**Figure 2:**
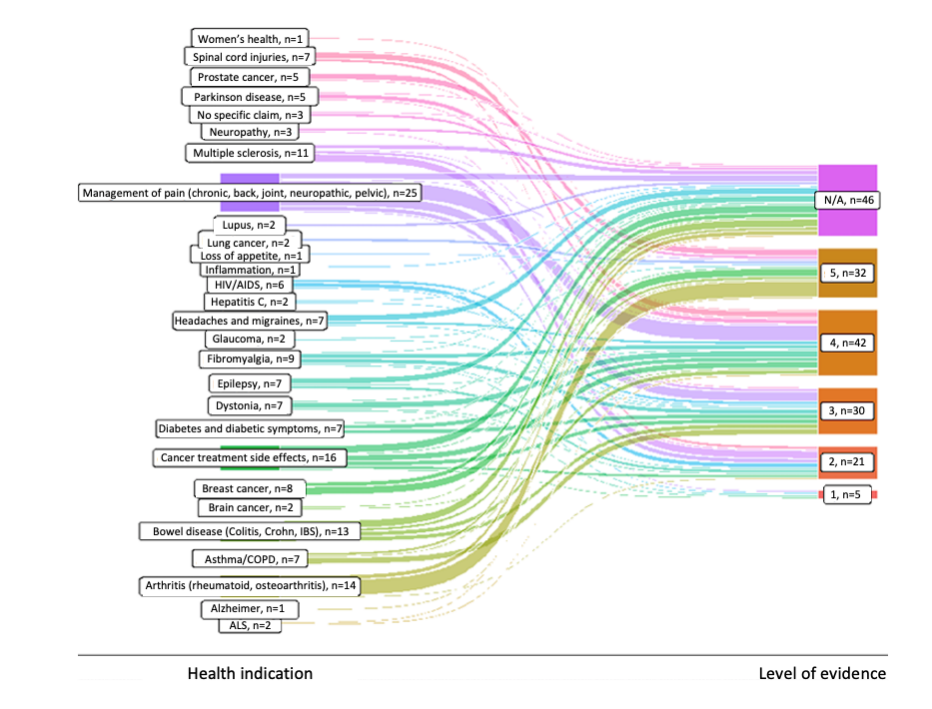
Levels of evidence for physical indications. ALS: amyotrophic lateral sclerosis; COPD: chronic obstructive pulmonary disease; IBS: irritable bowel syndrome; N/A: not applicable.

**Figure 3:**
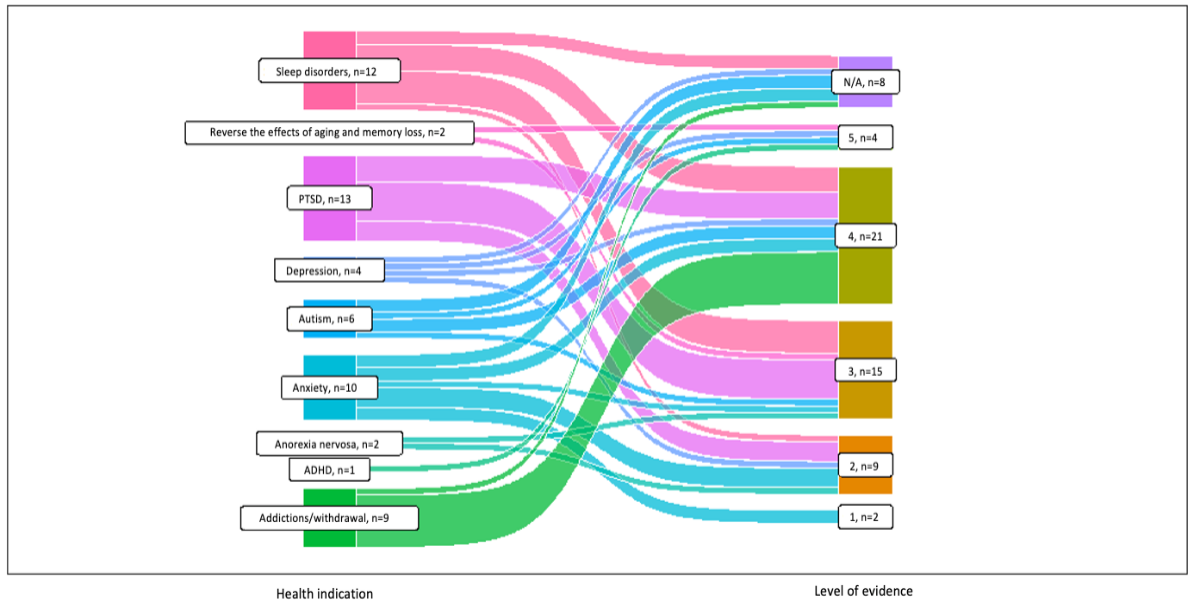
Levels of evidence for mental health indications. ADHD: attention deficit hyperactivity disorder; N/A: not applicable; PTSD: posttraumatic stress disorder.

Many studies (90/235, 38.3%) either were level 5 (36/235, 15.3%) or uncategorized (54/235, 22.9%) because they were nonsystematic, narrative reviews, or basic science studies. These classifications were split between studies targeting physical or mental illnesses for ease of display (Figures 2 and 3, respectively). A total of 7 of 235 (3%) studies were considered “high-level” level 1 studies (all systematic reviews), 30 of 235 (12.8%) studies were level 2 studies (RCTs), 45 of 235 (19.1%) studies were level 3 (non–randomized controlled cohort, cross-sectional studies or poorly conducted RCTs), and 63 of 235 (26.8%) studies were level 4 (case series and case-control studies).

## Discussion

### Principal Findings

This cross-sectional study of Ontario cannabis clinic websites found that the evidence supporting the use of medical cannabis was of poor quality. Despite this, positive medical claims for cannabis use are stated on cannabis clinic advertising. Our findings are congruent with previous literature describing the type of overstated advertising used by cannabis companies: weak research is used to argue for the effectiveness of an intervention by advertising it as a rigorous and thorough scientific study [25,34,35]. Other research has noted that cannabis companies often partner with academic institutions and research organizations to better market their product as being related to research [34]. One commentary postulated that this type of deception is aimed at everyday cannabis users, who may be less trained at reading scientific studies compared with those working at the health agencies where these claims are being evaluated [35]. Moreover, compared with other drug companies, cannabis companies have been critiqued for lackluster funding for testing the safety of medical cannabis; for example, while one of the largest cannabis companies was spending approximately US $2.5 million that year on cannabis research, other biomedical companies allocated billions of dollars to develop a single medical product [35,36]. The relationship built between medical cannabis companies and consumers has been compared with that of the tobacco industry, where tobacco products are targeted toward daily users, despite these users being more likely to experience tobacco-related harms [37].

The breadth of conditions advertised to be treatable by the clinics is also concerning, primarily because Canadian guidelines for medical cannabis prescription describe potential uses of cannabis for only 3 conditions: refractory chronic pain, refractory nausea and vomiting caused by chemotherapy, and refractory spasticity among patients with multiple sclerosis; the guidelines discourage cannabis prescriptions outside of these circumstances [18]. Hence, the advertisement of cannabis toward health conditions not supported by evidence may foster unwanted adverse effects among patients, especially if a patient uses cannabis frequently and beyond recommended limits [7]. Given the quality of evidence identified in this study, it is apparent that the cannabis prescriptions proposed by many of these clinics are unlikely to be based on strong evidence-based research [10,17]. Our findings align with previous appraisal of the evidence base for cannabis effectiveness. For example, a systematic review of systematic reviews by Allan et al [17] on the effectiveness of medical cannabinoids identified high-quality evidence for only 3 indications: neuropathic pain, chemotherapy-related nausea and vomiting, and multiple sclerosis–related spasticity. They identified serious adverse events, such as development of psychosis [17], and found that the RCTs included in cannabis systematic reviews were at high risk of bias due to irregularities in factors like size, duration, blinding, inclusion and exclusion criteria, and result reporting [17].

It was striking that few clinics described the harms of medical cannabis, especially when there is evidence suggesting that cannabis may be more likely to cause harm to patients rather than benefit them [17,18]. Specifically, many cannabis-prescribing clinics in Ontario claim to treat conditions for which cannabis may not be safe. Prolonged cannabis use may in fact *increase* the risk of developing depression [38,39] and worsen anxiety [39], especially if cannabis use starts during adolescent development [39], but in our study, these were some of the most common indications for which it was recommended. Smoking was one of the most common routes of administration in cited studies (76/235, 32.3%) even though research indicates that cannabis smoke damages the lungs comparably to cigarette smoke and increases the risk of lung problems substantially over time [40]. Yet on the websites of the clinics, no warnings related to smoking medical cannabis were found.

Our work contributes to the literature by demonstrating the weaknesses in the medical cannabis–related research promoted in Ontario. It is our hope that this research will spur more critical use and assessment of medical cannabis–related research and potential harms, and ultimately lead to stronger protections for those who seek to use medical cannabis.

### Limitations

Although the legalization of cannabis was implemented concurrently across Canada, this study included only clinics in Ontario. This specific sample may limit the studies that were examined; however, the evidence cited by the clinics was not limited geographically. It is possible that other medical clinics in the country make different claims.

Furthermore, we did not verify whether each clinic had a licensed physician working with them. Instead, we assessed that clinics identified in the search could be included if there was a statement on the website that a physician was involved. We found that, in general, websites did not name a specific physician and decided to be expansive with inclusion. We acknowledge that it is possible that some of these clinics may have stated that a physician was involved when that was not actually the case. However, we think this very unlikely because physician services in Ontario are fully paid for by the Ontario Health Insurance Plan at no out-of-pocket cost to the patient. It is therefore improbable that a clinic would report to have a physician but then not be able to provide that service.

Another limitation of this study design was our inability to generalize and summarize effect sizes for all cited papers. Although this was difficult, in part due to the extremely large number of studies cited and available in the full-text form (n=235), the studies themselves differed heavily in terms of methodology, specific aims, research populations, strain and type of cannabinoids used, and routes of administration.

Also, we implemented our search using Google, which may have given different search results to different members of the team. We limited bias by having 2 independent members search for cannabis-prescribing clinics in Ontario. Nevertheless, it is possible that some clinics may have been overlooked by our search strategy.

In addition, it is possible that using the term *pain* in our search criteria may have created a bias that led to pain being the most common indicator in our results. However, we think this is unlikely, as our search strategy did not change the underlying pool of results from which these data were drawn. Even if this biased our results to identify more clinics promoting the use of cannabis for pain, it would not have changed the results of the analysis of the strength of the evidence base.

### Conclusions

Cannabis clinic websites generally promote cannabis use as medically effective but cite low-quality evidence to support these claims and do not restrict claims to evidence-based indications. Most provide no information about potential harms, including the risks of smoking. When this is considered alongside the recreational uses of the same cannabis products, the broad introduction of cannabis as a general therapeutic is potentially misleading for medical practitioners and patients. Before using cannabis for therapeutic purposes, it is essential for patients and providers to consider the evidence (or lack thereof) for its effectiveness for a particular indication, as well as any potential benefits and harms associated with its use.

## Abbreviations

CBD: cannabidiol
RCT: randomized controlled trial
THC: tetrahydrocannabinol

## Data Availability

The data sets generated during and/or analyzed during this study are available from the corresponding author on reasonable request.
